# Heartbeat Cycle Length Detection by a Ballistocardiographic Sensor in Atrial Fibrillation and Sinus Rhythm

**DOI:** 10.1155/2015/840356

**Published:** 2015-07-01

**Authors:** Matthias Daniel Zink, Christoph Brüser, Patrick Winnersbach, Andreas Napp, Steffen Leonhardt, Nikolaus Marx, Patrick Schauerte, Karl Mischke

**Affiliations:** ^1^Department of Cardiology, Pneumology, Angiology and Intensive Care Medicine, University Hospital, RWTH Aachen University, Pauwelsstrasse 30, 52074 Aachen, Germany; ^2^Philips Chair for Medical Information Technology, RWTH Aachen, Germany

## Abstract

*Background.* Heart rate monitoring is especially interesting in patients with atrial fibrillation (AF) and is routinely performed by ECG. A ballistocardiography (BCG) foil is an unobtrusive sensor for mechanical vibrations. We tested the correlation of heartbeat cycle length detection by a novel algorithm for a BCG foil to an ECG in AF and sinus rhythm (SR).* Methods.* In 22 patients we obtained BCG and synchronized ECG recordings before and after cardioversion and examined the correlation between heartbeat characteristics.* Results.* We analyzed a total of 4317 heartbeats during AF and 2445 during SR with a correlation between ECG and BCG during AF of *r* = 0.70 (95% CI 0.68–0.71, *P* < 0.0001) and *r* = 0.75 (95% CI 0.73–0.77, *P* < 0.0001) during SR. By adding a quality index, artifacts could be reduced and the correlation increased for AF to 0.76 (95% CI 0.74–0.77, *P* < 0.0001, *n* = 3468) and for SR to 0.85 (95% CI 0.83–0.86, *P* < 0.0001, *n* = 2176).* Conclusion.* Heartbeat cycle length measurement by our novel algorithm for BCG foil is feasible during SR and AF, offering new possibilities of unobtrusive heart rate monitoring. This trial is registered with IRB registration number EK205/11. This trial is registered with clinical trials registration number NCT01779674.

## 1. Introduction

Heart rate control is of importance for patients suffering from atrial fibrillation (AF) [1] or heart failure [2] to improve morbidity and mortality. Heart failure is frequently found amongst the elderly and is often associated with arrhythmias like AF. Above the age of 60, the prevalence of AF is around 5–10%, with about 6 million Europeans and more than 3 million US Americans suffering from AF [3–5]. Up to 90% of AF episodes are paroxysmal, especially in its early stage, and up to 90% are asymptomatic [6, 7]. This is of great clinical relevance since AF is responsible for up to 30% of ischemic strokes [3], for systemic embolisms [8], and for an increased perioperative risk [9].

The gold standard for heart rate diagnosis is an ECG, but, for example in cases of asymptomatic and paroxysmal AF its diagnosis with intermittent ECG recordings is difficult. Recent evidence suggests that continuous ECG monitoring with implantable pacemakers can detect all relevant AF episodes [10], but, due to the large number of patients at risk, implantable monitoring devices are not affordable. Furthermore, prolonged regular ECG monitoring seems more effective in detecting silent AF episodes than short-term continuous ECG recordings [11]. For this reason the National Heart, Lung & Blood Institute Expert Panel of the United States of America encourages the development of new methods and technologies for asymptomatic AF detection [12]. New devices such as smartphone applications [13] and wearable [14] and videoplethysmographic sensors [15] are being tested as potential candidates, but to date their clinical application remains difficult.

In this original investigation, we used a ballistocardiographic sensor in a prospective cohort of patients with AF receiving an electric cardioversion. The sensor can be positioned beneath conventional textiles and bed sheets and measured the mechanical equivalent of the heartbeat indicating bradycardia, tachycardia, and arrhythmia by the calculated cycle length.

The aim of this study was to evaluate the correlation of the heartbeat analyzed by our novel algorithm compared to an ECG as the gold standard.

## 2. Materials and Methods

22 patients with AF scheduled for elective electric cardioversion at the University Hospital of Aachen were recruited. After informed consent, all patients were enrolled according to the following inclusion criteria: presence of AF with scheduled, elective cardioversion and at least 18 years of age. Exclusion criteria were pregnancy or lactation, mental incapacitation, or implanted electric device. Baseline demographic, clinical, laboratory, ECG, and synchronized BCG data were collected before and after electric cardioversion by trained staff. All recordings were performed in a supine position in spontaneously breathing participants.

Electric cardioversion was performed by a trained physician of the department of cardiology after exclusion of left atrial auricular thrombus by transoesophageal echocardiography.

The study was performed between January 2012 and March 2013 at the Department of Cardiology, Pneumology, Angiology and Intensive Care Medicine, University Hospital RWTH Aachen, Germany. The clinical trial was approved by the Ethics Committee of the Medical Faculty of the University Hospital Aachen (registration number: EK205/11, date: 27 May 2011; ClinicalTrials.gov: NCT01779674) and met current legal requirements (German Medical Devices Act and Code of Medical Ethics) as well as ethical principles contained in the Declaration of Helsinki and Good Clinical Practice guidelines.

### 2.1. Data Collection

For data collection a dedicated measuring cart with an “IntelliVue MX800 Patient Monitor” (Koninklijke Philips N. V., Amsterdam, Netherlands) connected to a personal computer was purpose-built. The electrical integrity was approved by the VDE (Verband Deutscher Elektrotechnik Elektronik Informationstechnik e.V., Frankfurt, Germany) for EN IEC 60601-1. For electronic data management an electronic case report form was programmed in OpenClinica (OpenClinica, LLC, Waltham, MA, USA).

### 2.2. Ballistocardiographic Sensor

**Figure 1:**
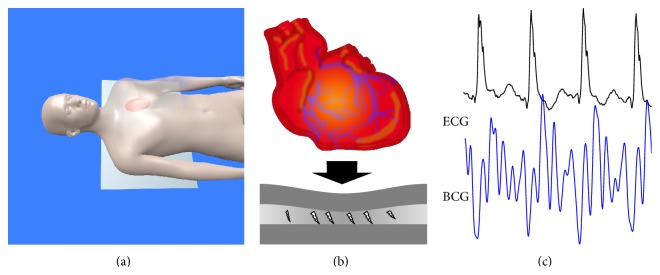
Heartbeat measurement by a BCG foil: (a) the BCG sensor foil is positioned under the chest of the patient in a supine position; (b) mechanical contraction of the heart induces impedance change on the BCG sensor foil; (c) a BCG (blue signal) related signal is calculated and synchronized to an ECG (black signal).

Ballistocardiography (BCG) is a technique to monitor mechanical activity of the heart by recording mechanical forces on the body's surface [16]. The basic concept has been known since the 19th century [17]. However, recent advances in sensor technologies have allowed the integration of highly sensitive mechanical sensors into beds for the purpose of unobtrusive cardiac monitoring [18, 19]. We used a thin and flexible foil, consisting of charged polymer layers containing air voids that behave in a similar way to electrical capacitors. Mechanical activity causes physical deformations of the sensor's geometry. If the geometry of the enclosed air voids changes, their electrical charges move with respect to each other. These charge shifts can be measured by the sensor electrodes, converted to a voltage signal, and subsequently displayed as an ECG related signal (Figure 1).

### 2.3. Heartbeat Measurement by BCG Sensor

Every patient was measured in a supine position on a mattress with an attached BCG foil. BCGs were recorded by a ballistocardiographic sensor (Emfit Ltd., Vaajakoski, Finland). The sensor foil (30 × 60 cm) was positioned under the textile bed sheet and was invisible to the patients (Figure 1(a)). The motion signal was recorded by the sensor foil along a dorsoventral axis. There was no direct contact between the ballistocardiographic sensor and the patient.

### 2.4. Signal Processing

**Figure 2:**
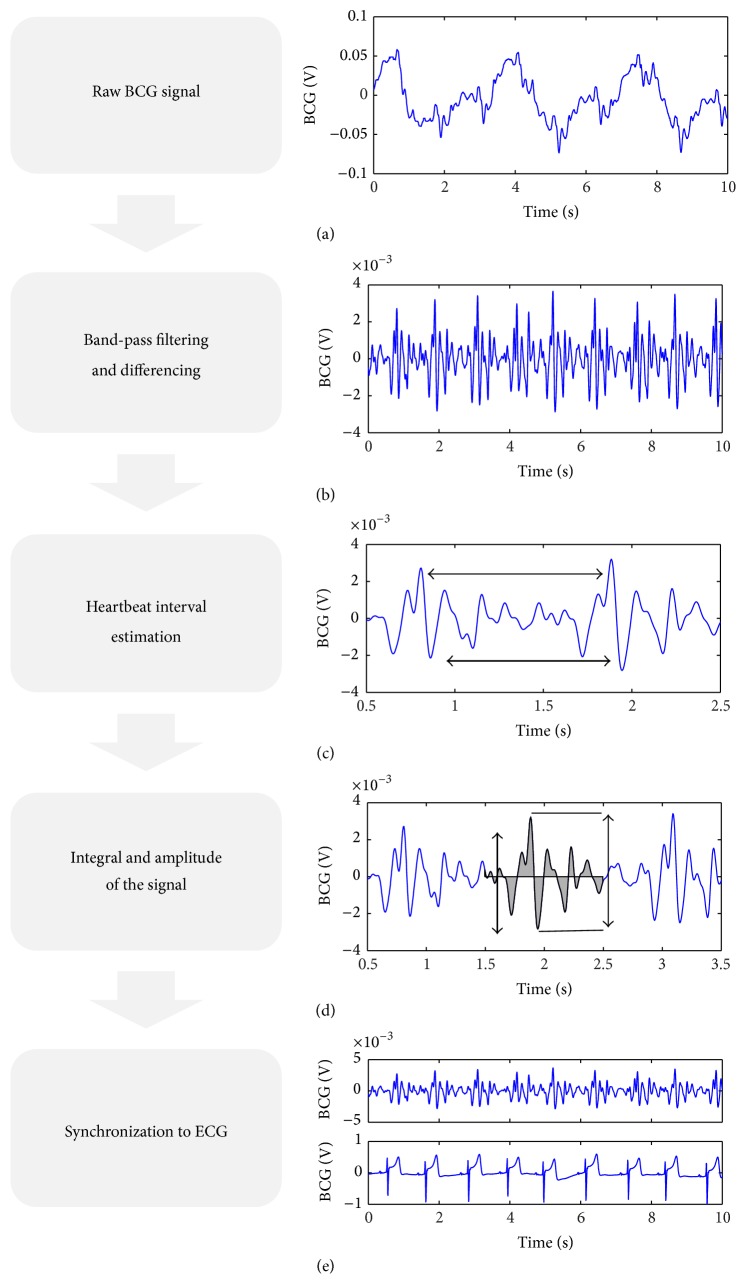
Signal processing of BCG data: (a) the raw signal includes in its highest deflections inhalation and exhalation; (b) after time-domain filtering the breathing component is removed and repeating oscillations as a surrogate for the heart contraction are visible; (c) the local interval estimator defines the cycle length by beat-to-beat analysis (Figure 3); (d) additional calculations for the integral of the BCG complex and the maximal amplitude deflections are carried out; (e) the BCG signal is synchronized to the ECG.

**Figure 3:**
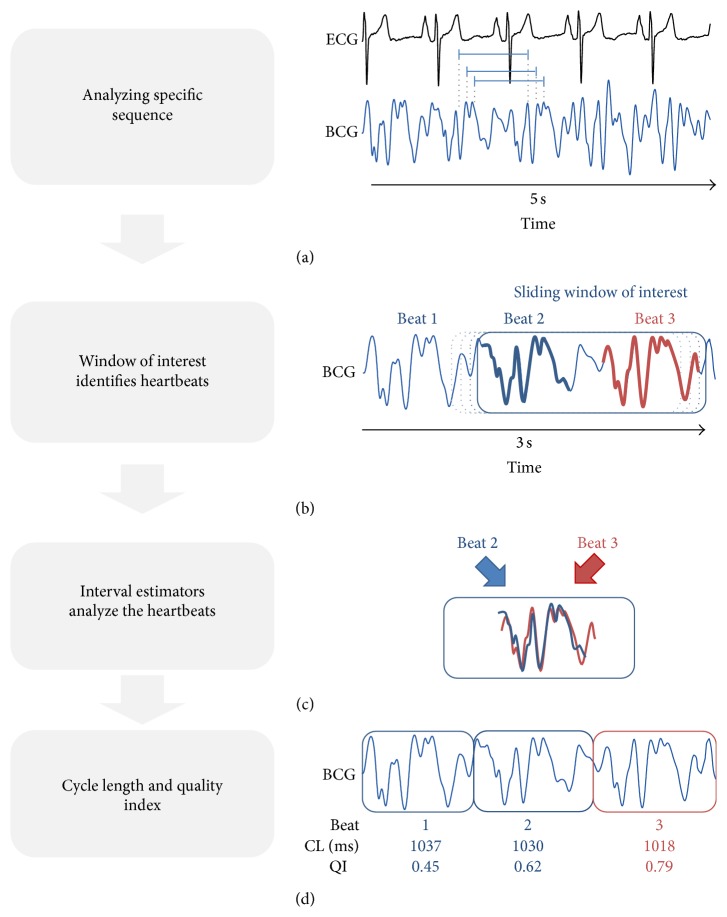
Estimating the heartbeat cycle length: (a) the window of interest analyzes the signal for repeated amplitude patterns and estimates the cycle length; (b) a sliding window of interest performs basic threshold measurements and identifies two consecutive heartbeats; (c) three local interval estimators analyze the signals and each estimates a cycle length: the match between the three estimators is the quality index; (d) the window of interest moves forward and the estimated cycle length and quality index are displayed. ^*^BCG: ballistocardiogram; CL: cycle length; QI: quality index.

The BCG sensor acquired mechanical movement by a change of charge with 1000 Hz. The calculating time allowed an almost real-time analysis with a latency of <2 seconds. Heart contraction (Figure 1(b)), valve movement, blood flow, respiration, muscular activity [20, 21], and other mechanical activities were measured by the BCG foil and were part of the resulting BCG signal (Figure 1(c)). Depending on the subject's position related to the sensor, the force vector of each mechanical activity produced corresponding amplitudes. The superposition of different mechanical vectors impaired the signal analysis, so that the genuine signal had to be cleaned by filtering for the specific frequency range in question. A genuine BCG signal (Figure 2(a)) measured in a dorsoventral direction showed, along its vertical axis, slow oscillations for about 5 seconds of breathing which included smaller deflections oscillating at a higher frequency. By time-domain filtering and differencing, the breathing component was removed and the smaller, higher frequency oscillations became visible (Figure 2(b)). For filtering we used fixed and identical filters for all recordings with a cutoff frequency of 0.5 Hz and 80 dB stop-band attenuation. By a beat-to-beat analysis of local interval estimators the cycle length was calculated (Figure 2(c); Figure 3). Additional calculations such as quality index, integral, and maximal amplitude of the BCG complex were performed afterwards (Figure 2(d)). In the final step the BCG data was harmonized to a synchronized recorded ECG (Figure 2(e)). In this step the BCG peaks showed a specific sequence corresponding to the recorded ECG (Figure 2(e)).

### 2.5. Cycle Length Detection and Quality Index

Common techniques for automated heartbeat analysis consisted of locating relevant events, like the QRS complex, to obtain beat-to-beat intervals. Prior knowledge of the characteristics for the events of interest was necessary. Due to the variability of the inter- and intrasubject BCG deflection depending on the vector of interest related to the sensor and artifacts, these kinds of algorithms did not seem applicable for beat-to-beat analysis using the BCG signal [22]. We used a novel approach for heart rate analysis inspired by the so-called pitch-tracking method for speech processing [23].

**Figure 4:**
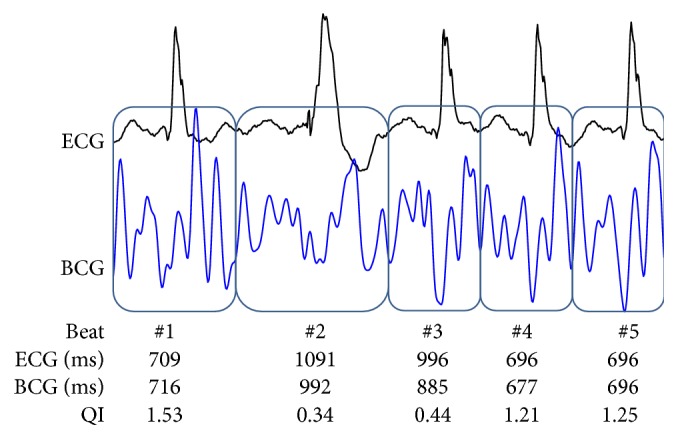
ECG (black signal) synchronized BCG (blue signal), heartbeat count, ECG cycle length, and corresponding estimated BCG cycle length and quality index are simultaneously displayed. Beat 2 is a premature ventricular contraction resulting in a minor accordance of ECG and BCG cycle length. Also heartbeat 3 is affected by premature ventricular contraction; the following heartbeats show near-perfect accordance to the ECG cycle length with a high corresponding quality index. ^*^BCG: ballistocardiogram; QI: quality index.

The first window of interest was of constant size by a prior defined frequency of interest. The specific sequence of a heartbeat was not known but the assumption was that consecutive heartbeats consisted of a corresponding sequence of amplitudes. The algorithm analyzed the BCG signal for repeated patterns of deflections and identified these events as heartbeats (Figure 3(a)). A sliding window of interest moved 200 ms forward and an adaptive threshold measurement was performed of the window location. If the thresholds were violated, the presence of a high-energy artifact was assumed. The location was marked as corrupted and the algorithm restarted. In the case of no threshold violation the algorithm continued. The window of interest was more than twice the length of the estimated cycle length and identified two consecutive heartbeats for their specific amplitude pattern (Figure 3(b)). Three local interval estimators compared the isolated sequences to each other and each of them estimated a cycle length. The quality index defined the match of these three local interval estimators. The higher the accordance between the three estimators, the higher the quality index and the more precise the calculated cycle length (Figure 3(c)). Finally the cycle length and the corresponding quality index were defined and the window of interest moved on (Figure 3(d)). After computing the algorithm the results were displayed in less than two seconds (Figure 4).

By means of the quality index it was possible to identify artifacts or hampered signals by filtering the whole recorded signal for a specific quality. Subsequently, we added the synchronized ECG signal and analyzed the beat-to-beat interval in ECG using the “Open Source Arrhythmia Detection Software” (EP Limited, 35 Medford St., Somerville, MA, USA). At least 10 consecutive heartbeats were used for signal analysis.

### 2.6. Statistical Analysis

For correlation analysis we used Pearson's correlation coefficient and the Bland-Altman Plot for visual analysis. For qualitative analysis all values are expressed in percentages and absolute numbers. Values of *P* < 0.05 were considered as statistically significant. Statistical analysis was performed with SPSS 21 (BM Corp., Released 2012, IBM SPSS Statistics for Windows, Version 21.0., Armonk, NY, IBM Corp.) and MedCalc Statistical Software version 13.3.1 (MedCalc Software bvba, Ostend, Belgium; http://www.medcalc.org; 2014).

## 3. Results

The average age of patients was 72; 75% were male. Participants had a significantly higher heart rate before electric cardioversion (AF 88 ± 21 versus SR 67 ± 21 beats per minute). Two participants suffered serious medical problems during cardioversion so that due to many artifacts and a short measuring time these data were excluded. One patient converted spontaneously to sinus rhythm (SR) prior to cardioversion and his data were included in the SR group only. After successful cardioversion two patients showed premature ventricular beats after every normal sinus heartbeat (bigeminus) and thus were not considered as SR data. In five patients cardioversion was not successful; therefore their data after cardioversion were included in the AF group. Overall, we analyzed the data of 20 patients.

Cardioversion converted AF to SR and increased the mean ECG cycle length significantly (*P* < 0.001) from 729 ± 280 ms to 1004 ± 180 ms. Comparably, the mean BCG cycle length increased significantly (*P* < 0.001) from 758 ± 276 ms to 983 ± 199 ms (Table 1). After cardioversion, the BCG amplitude and integral of BCG complex decreased significantly (*P* < 0.0001) with a narrow standard deviation indicating a more consistent heartbeat signal complex in BCG during SR (Table 1).

**Figure 5:**
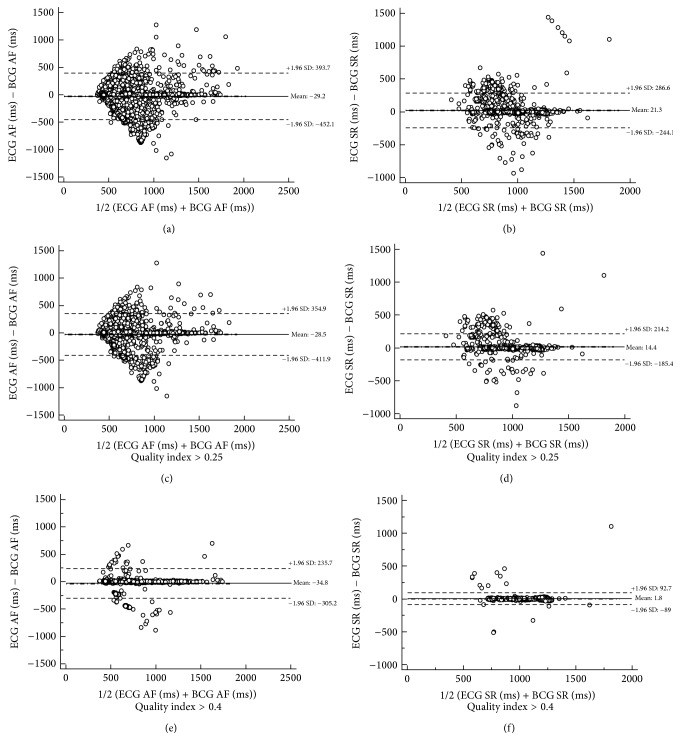
Correlation of analyzed cycle length of ECG and BCG in different quality index steps. Left AF, right SR (Bland-Altman Plot: *y*-axis: mean of difference ECG−BCG and 95% limits of agreement ±1.96 ^*^SD): (a) all analyzed AF data; (b) all analyzed SR data; (c) AF data filtered by quality index >0.25; (d) SR data filtered by quality index >0.25; (e) AF data filtered by quality index >0.4; (f) SR data filtered by quality index >0.4.

We analyzed 4317 heartbeats between BCG and ECG during AF resulting in a correlation coefficient of 0.7 (0.68–0.71, *P* < 0.0001, *n* = 4317) (Figure 5(a)). 2445 heartbeats during SR were analyzed; here we found a correlation coefficient between BCG and ECG of 0.75 (95% CI 0.73–0.77, *P* < 0.0001, *n* = 2445) (Figure 5(b)). By filtering the AF signal with the quality index >0.25 (Table 2), the number of analyzable heartbeats was reduced to 80%, and the correlation coefficient increased to 0.76 (95% CI 0.74–0.77, *P* < 0.0001, *n* = 3468) (Figure 5(c)). The correlation in SR increased to 0.85 (95% CI 0.83–0.86, *P* < 0.0001, *n* = 2176) (Figure 5(d)) by filtering with the quality index >0.25, with 89% of heartbeats remaining analyzable data. For higher quality indexes the resulting correlation coefficient increased with a decrease of analyzable heartbeat intervals. Thus, a quality index >0.4 resulted in a high correlation coefficient during AF with 0.89 (95% CI 0.88–0.90, *P* < 0.0001, *n* = 1606) (Figure 5(e)) and a near-perfect correlation coefficient of 0.95 (95% CI 0.95–0.96, *P* < 0.0001, *n* = 1410) (Figure 5(f)) during SR.

**Figure 6:**
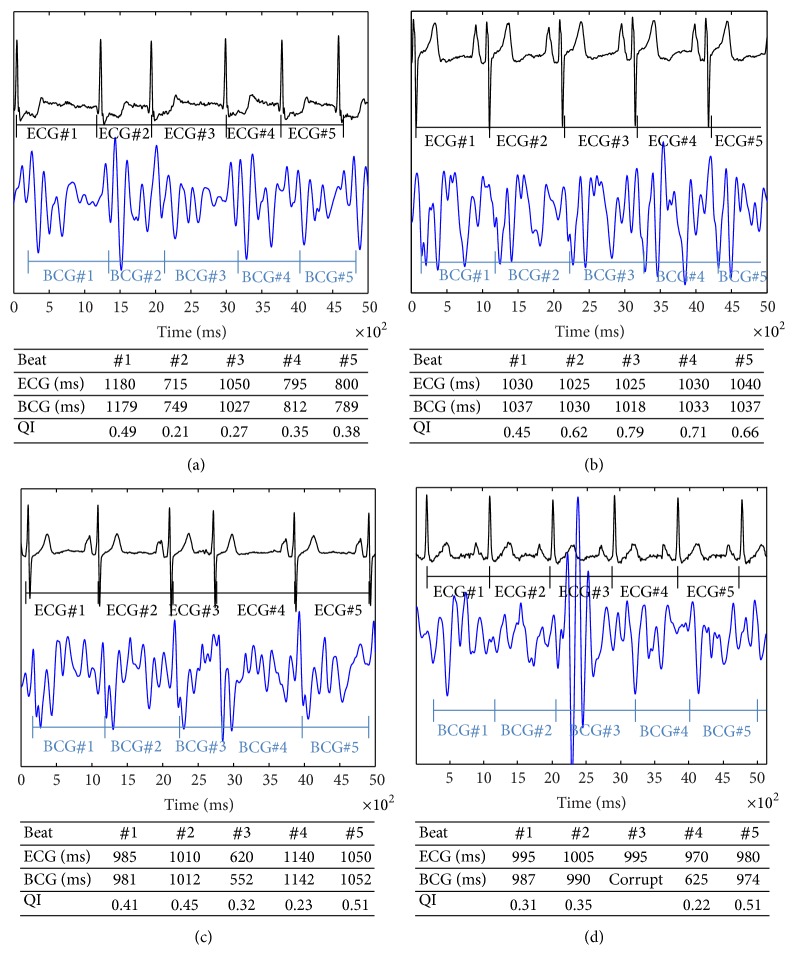
Examples of cycle length detection by synchronized ECG and BCG with corresponding quality index. ECG (black signal) and synchronized BCG (blue signal). (a) AF shows a good correlation of the ECG and synchronized BCG interval detection. BCG#2 indexing a change in heartbeat cycle length resulting in an inaccurate BCG cycle length detection with corresponding decreased quality index. (b) SR after cardioversion with a near-perfect ECG and BCG cycle length correlation resulting in a high quality index for each heartbeat above 0.4. (c) SR after cardioversion with a premature atrial contraction (BCG#3). The corresponding quality index indicates a poor BCG quality for the premature contraction (BCG#3) and the following beat (BCG#4) due to a change in the deflection pattern and a good quality of BCG signal in normal SR. (d) SR after cardioversion with a good BCG cycle length detection interrupted by a high-energy artifact, most likely a moving artifact with a BCG interval marked as corrupt (BCG#3). The consecutive beats are all detected with an improving ECG and BCG cycle length correlation and an increasing quality index. Although the BCG pattern changed after the moving artifact no training phase was necessary for cycle length detection. ^*^BCG: ballistocardiogram; QI: quality index.

Figures 6(a)–6(d) document examples of BCG analysis corresponding to quality index and synchronized ECG. For AF with normal heart rate (Figure 6(a)) the BCG interval detection showed a good correlation to ECG. In heartbeats with inaccurate correlation of BCG and ECG cycle length the corresponding calculated quality index of the BCG signal decreased too.

In SR (Figure 6(b)) after cardioversion the ECG and BCG cycle length correlation was near perfect resulting in a high quality index for each heartbeat.

Premature atrial contraction in SR (Figure 6(c)) after cardioversion resulted in a decreased BCG quality index and poor correlation to the ECG for the premature contraction and the following heartbeat. However, a good correlation of BCG signals was observed during normal sinus beats (without premature contraction) with a corresponding high quality index.

Figure 6(d) depicts the effect of a movement artifact. The match between BCG and ECG was interrupted by a high-energy artifact resulting from patient movement. The episode was marked as corrupt and could be used for filtering the BCG signal. After the artifact the first heartbeat showed a poor correlation of cycle length detection due to a changed amplitude pattern. However, the algorithm needed no training phase and the consecutive heartbeats showed again a good cycle length correlation and an improving quality index.

## 4. Discussion

The present study demonstrates that heartbeat interval detection by a ballistocardiographic sensor during SR and AF is feasible using a novel pitch-tracking inspired algorithm. Heartbeat analysis is currently mainly performed by ECG or by photoplethysmographic sensors. The ECG represents the gold standard for heart rate measurement. Although new sensor technologies are the focus of research to deal with the upcoming problems of an aging society and an increasing demand for outpatient diagnostic tools, none have proven clinically useful so far. The pitfalls of these new sensor technologies are compliance of the patient, operability, availability, and accuracy.

In our study participants were placed in a supine position with their chests above the BCG foil. The sensor is unobtrusive and has no direct contact to the skin. This offers the possibility of integrating the sensor foil into any bed sheet. Other measuring situations such as a prone or sideways position are theoretically possible. In particular, the prone position might offer a better signal for the heartbeat analysis owing to direct contact of the BCG foil and the apical impulse of the heart. However, this position was not tested due to the study setting of cardioversion.

There seems to be a circadian distribution of arrhythmias with peaks at different times during the day [24, 25]. Thus the proposed technology, which can be easily integrated into a mattress, may potentially be suited for large scale and long-term recording of the heart rate and rhythm during sleep. The measurement system needs the BCG foil and a computer for the algorithm. Excluding the attached computer, the costs for the system remain below $100.

The BCG signal measures any mechanical vibration. To receive the best results the longitudinal axis of the movement of interest should be positioned perpendicular to the measuring foil and other movements have to be excluded because the signal is hampered by any other movement which puts pressure on the foil. In real life conditions this will not be possible so a robust and flexible algorithm is needed to exclude artifacts and filter the signal of interest. Due to the ambiguous nature of the BCG deflection our approach does not search for a specific or defined signal appearance but looks for repeating signal deflections. For this reason no training is needed and a change of BCG deflection, for example, after a body movement, does not affect the analysis. In clinical practice patients are advised to remain motionless during ECG recording; this would probably also improve the BCG signal quality but was not tested. The algorithm at this point does not offer a qualitative analysis of the heartbeat characteristics and is not able to distinguish between SR and AF.

In contrast to photoplethysmographic sensor technologies, BCG measures the mechanical movements of the organ of interest. Peripheral pulse deficits due to low blood pressure, increased peripheral resistance, venous return, sympathetic arousal, temperature, or centralization of circulation do not interfere with the signal as much as they do for the photoplethysmographic sensors [15]. Due to different filling conditions and an irregular heartbeat during AF the match between consecutive heartbeats in the BCG signal alternates. Thus, arrhythmia heartbeat detection is challenging for the algorithm and resulted in a decreased quality index (0.41) during AF in contrast to sinus rhythm (0.52) as described in Table 1. The lower quality index during AF or premature ventricular contraction compared to sinus rhythm could hamper the recognition and differentiation of true heartbeats in contrast to artifacts. However, we were able to calculate cutoffs for the quality index to differentiate between quality index values during AF and quality index values during artifacts. Thus, the algorithm remains robust in its signal detection under different filling conditions and motion sequences of the heart such as during AF or premature ventricular contractions (Figures 6(a)–6(d)).

We have seen encouraging results with a good baseline correlation of the BCG signal to the synchronized ECG. The algorithm needs no training for heartbeat detection and offers almost real-time cycle length analysis with a delay of less than 2 seconds. So in addition to the recording opportunities like a Holter ECG a bedside application seems possible too. Interestingly and in contrast to our own previous results, the baseline BCG signal during SR offers a lower quality index and correlation coefficient than expected. This is caused by the direct recording after cardioversion during the awaking period in which there is some body movement. These movement artifacts could be filtered easily by the quality index, resulting in a high correlation coefficient. In contrast to our expectations, the BCG signal also offers good interval recognition during AF even though different filling conditions and a beat-to-beat change of cycle length during AF can hamper the BCG signal. This shows the strength and flexibility of the used algorithm.

### 4.1. Limitations

The number of patients included in this feasibility study was low. However, the number of heartbeats analyzed in the study was high. The filter includes means to filter the organ and frequency of interest and distinguish artifacts so the algorithm works in the frequency we are interested in (for this investigation from 30 to 180 beats per minute). Other cycle lengths could have been neglected but were not present during the data collection. In addition, the algorithm presented in the study provides no qualitative assessment of the rhythm so a differentiation between SR and AF is not presented to the user. However, the aim of the study was not to distinguish between SR and AF but to assess the feasibility of cycle length analysis during SR and AF.

## 5. Conclusion

In conclusion, we demonstrated that the heartbeat cycle length detection by our novel algorithm with a ballistocardiographic sensor is feasible in AF and SR with a good correlation to a synchronized ECG. Artifacts can be filtered by using a quality index of each analyzed heartbeat in the BCG signal.

## Figures and Tables

**Table 1:** ECG and BCG interval characteristics before and after cardioversion.

	Atrial fibrillation	Sinus rhythm	*P* value
	Mean (±SD)	Mean (±SD)	
ECG interval [ms]	729 (±280)	1004 (±180)	<0.001
BCG interval [ms]	758 (±276)	983 (±199)	<0.001
Quality index [AU]^*^	0.41 (±0.21)	0.52 (±0.27)	<0.001
BCG amplitude [AU]^*^	0.088 (±0.047)	0.059 (±0.03)	<0.001
Integral BCG complex [AU]^*^	0.018 (±0.011)	0.011 (±0.006)	<0.001

^*^AU: arbitrary units.

**Table 2:** Filtering of the measured BCG during AF and SR by the quality index with remaining analyzable episodes and corresponding correlation coefficient.

Quality index	Atrial fibrillation				Sinus rhythm			
	*n*	%	*r*	95% CI	*n*	%	*r*	95%
>0.1	4317	100	0.70	0.68 to 0.71	2445	100	0.75	0.73 to 0.77
>0.15	4301	100	0.70	0.69 to 0.72	2440	100	0.76	0.74 to 0.78
>0.2	4071	94	0.72	0.71 to 0.74	2359	96	0.79	0.77 to 0.80
>0.25	3468	80	0.76	0.74 to 0.77	2176	89	0.85	0.83 to 0.86
>0.3	2711	63	0.83	0.82 to 0.84	1933	79	0.901	0.9 to 0.92
>0.35	2088	48	0.87	0.86 to 0.88	1670	68	0.94	0.93 to 0.94
>0.4	1606	37	0.89	0.88 to 0.90	1410	58	0.95	0.95 to 0.96

## Abbreviations

AF: Atrial fibrillation
AU: Arbitrary units
BCG: Ballistocardiogram.
